# Disease Progression, Clinical Features, and Risk Factors for Pneumonia in Unvaccinated Children and Adolescents with Measles: A Re-Emerging Disease in Romania

**DOI:** 10.3390/ijerph192013165

**Published:** 2022-10-13

**Authors:** Mirela Turaiche, Mirela Loredana Grigoras, Felix Bratosin, Iulia Bogdan, Adrian Vasile Bota, Bianca Cerbu, Camelia Vidita Gurban, Prima Hapsari Wulandari, Srivathsava Gurumurthy, Kakarla Hemaswini, Cosmin Citu, Iosif Marincu

**Affiliations:** 1 Methodological and Infectious Diseases Research Center, Department of Infectious Diseases, “Victor Babes” University of Medicine and Pharmacy, 300041 Timisoara, Romania; 2 Department of Anatomy and Embryology, “Victor Babes” University of Medicine and Pharmacy, Eftimie Murgu Square 2, 300041 Timisoara, Romania; 3 Department of Biochemistry, “Victor Babes” University of Medicine and Pharmacy, Eftimie Murgu Square 2, 300041 Timisoara, Romania; 4 Massachusetts General Hospital, Harvard Medical School, 55 Fruit St, Boston, MA 02114, USA; 5 Mysore Medical College and Research Institute, Irwin Road, Mysuru 570001, India; 6 Malla Reddy Institute of Medical Sciences, Suraram Main Road 138, Hyderabad 500055, India; 7 Department of Obstetrics and Gynecology, “Victor Babes” University of Medicine and Pharmacy, Eftimie Murgu Square 2, 300041 Timisoara, Romania

**Keywords:** measles, infectious disease, public health

## Abstract

Measles causes in vaccinated children, with some exceptions, a mild disease, while the unvaccinated can suffer complications that result in serious consequences and even death. Although the introduction of the measles vaccine has reduced the number of cases and the viral spread, the current downward vaccination trend has resulted in the resurgence of the disease. Currently, Romania has a measles vaccination coverage below the 95% safety threshold. Thus, an outbreak started in 2016 and still ongoing in Romania, many cases being identified in the Western region in the pediatric population. Our objective was to conduct a thorough examination of clinical characteristics, evolution, and risk factors in vaccinated and unvaccinated children in this region. To reach our objectives we used a retrospective cohort analysis. The authors reviewed clinical and laboratory data from patients hospitalized at “Victor Babes” Hospital for Infectious Diseases and Pulmonology in Timisoara. We found a total of 136 qualifying cases of measles among the children admitted to this facility. The two comparison groups consisted of 104 children under 10 years and 32 patients between 10 and 18 years. An important characteristic of both study groups was the high prevalence of patients from the Roma ethnicity, which, although represents a minority in Romania, the prevalence was over 40% in the current study. The infection source was in 40.4% of children under 10 years inside the family, while 71.9% of infections in the group of adolescents were isolated (*p*-value = 0.047). The multivariate risk factor analysis identified as independent risk factors for the development of pneumonia the older age of patients (OR = 1.62), poor nutritional status (OR = 1.25), Roma ethnicity (OR = 2.44), presence of anemia (OR = 1.58), and procalcitonin (OR = 3.09). It is essential to handle these risk factors in a patient with measles, especially in conjunction with an unknown vaccination status. To achieve a vaccination rate greater than 95 percent for Romanian children, measles vaccination awareness must be promoted, moreover in the Roma population. More comprehensive preventative methods must be developed promptly with the objective of eradicating measles in Romania via a vigorous vaccination campaign.

## 1. Introduction

Measles is a highly infectious virus-borne illness caused by Morbillivirus of the family Paramyxoviridae. When someone coughs or sneezes, the virus is spread by droplets [1]. The most contagious period is considered the prodrome that lasts 3–5 days after rash onset, although it can extend further because measles RNA can be present in blood, urine, and nasopharyngeal mucosa for extended periods of time, even months following the onset of the rash. The infectious period is greatest during the phase during which the virus replicates primarily in the upper respiratory tract rather than the lower respiratory tract [2]. Measles is defined by cyclical epidemics, which occur primarily in the winter in correlation with social risk factors such as crowded enclosed spaces, school gatherings, etc. The measles epidemics also seem to be characterized by longer cycles of several years due to an increasing trend in susceptible non-immunized people during previous outbreaks [3]. According to much research, another significant element affecting the cyclicality and length of epidemics is the birth rate [4].

Measles has an incubation period of around 10–14 days, beginning four days before the rash appears and ending four days after it appears [5]. The elevated levels of viremia linked with the period of more violent coughing coryza, that produce more droplets and increase the intensity of viral transmission [6]. However, the virus can only be maintained in human populations through unbroken transmission chains since it does not induce known infections in animals, nor it is detectable in animal reservoirs [7]. Uncomplicated diseased patients may recover rather quickly and without complications or adverse effects. However, some of the following disease complications may occur: encephalitis (1 case in 1000) [8], middle ear infections [9], and measles-related pneumonia or secondary bacterial pneumonia affecting up to 60 in 1000 patients, and [10,11,12].

Measles is no longer considered only a childhood disease [13], as it may occur at any age. However, individuals over the age of 20 are at a higher risk of developing complications, moreover, if the patient was not vaccinated or fully vaccinated, or when vaccination coverage is lower than the minimum required 95% to prevent viral spread [14]. Recent vaccination strategies have pushed the age structure of measles infection towards adolescence and adulthood in populations who have received two doses of measles vaccine [15]. Along with this shift in the epidemic slope by age, the number of measles outbreaks has multiplied in areas with low vaccination coverage and in healthcare settings with vulnerable communities not protected by vaccination, such as the very young, and patients with multiple comorbidities [16].

Between 1980 and 2015, Romania documented a small number of cases of measles after the introduction of the measles immunization into the National Vaccination Program [17]. Since 2005, the Romanian immunization campaign has included two doses of MMR trivaccine at 12 months and 6–7 years of age. Romania has seen a large spike in measles infections since 2015 as a consequence of a considerable decline in the number of people willing to vaccinate [18]. Consequently, a measles outbreak was proclaimed in 2016, and an extra dosage of MMR trivaccine became obligatory at the age of 9 months. According to the official percentages of measles vaccination coverage reported in Romania in 2019, 76% of the vaccination-targeted population had received the second dose of measles vaccine, while 65.4% of the target population vaccination had received two doses of measles vaccine, resulting in a proportion of 90.9% of the target population being protected against measles [19]. After four years of administration on this schedule, beginning in August 2020, the extra dosage of MMR delivered during childhood will be stopped [20]. Thus, we planned to study the Romanian pediatric population four years after the governmental change in the vaccination schedule in the western region that encountered most of the measles cases during the 2016 epidemic. Our aim was to provide a comprehensive look into pediatric clinical features, evolution, and risk factors for developing pneumonia, stratified by patients’ age group in children and adolescents.

## 2. Materials and Methods

### 2.1. Study Design and Ethics

The authors performed a retrospective cohort analysis to track the features and effects of measles virus infection in the pediatric population under 18 years of age. We analyzed clinical and analytical data from patients admitted to “Victor Babes” Hospital for Infectious Diseases and Pulmonology from Timisoara. The patients were selected from 1 January 2019 to 1 January 2020. The sample size and key features were identified via the use of a population-based administrative database of patients in the inpatient setting over the study period. Our comprehensive database included patient medical records that were protected by privacy laws and gathered with the patient’s permission. The patient’s demographics, medical history, laboratory profile, and management were included. The baseline characteristics and procedures of all patients were recorded in the hospital database and in paper patient records inspected by certified clinicians participating in the current inquiry. In the children population hospitalized in our hospital, we detected a total of 136 cases of measles.

The Local Commission of Ethics for Scientific Research at the “Dr. Victor Babes” Clinical Hospital for Infectious Diseases and Pulmonology in Timisoara is governed by the provisions of Article 167 of Law No. 95/2006, Article 28 of Order 904/2006, and the EU Good Clinical Practice Directives 2005/28/EC, the International Conference on Harmonization of Technical Requirements for the Registration of Pharmaceuticals for Human Use (ICH), and the Declaration of Helsinki—Recommendations Guiding Mediation. On 15 December 2021, the present research was accepted with approval number 12572. By completing an informed consent form, each parent agreed to provide children with private medical data.

### 2.2. Patient Inclusion and Variables

The main inclusion condition required patients to be below the age of 18. Additionally, patients were included if they were hospitalized with a proven measles infection by positive lab result or following the clinical classification as the Center for Diseases Control (CDC) declared in 1983: “(1) a generalized maculopapular rash lasting 3 or more days, (2) temperature of 38.3 C (101 F) or greater, and (3) one of the following: cough, coryza, conjunctivitis” [21]. The measles infection was also confirmed through the IgM antibody detection. Patients were excluded from the research if their personal data were insufficient or if permission to use their medical data was not granted by their legal guardians’. Age stratification was performed to separate our data into two comparison groups, children and adolescents. An adolescent was considered by the definition of the United Nations (UN) convention as a child aged between 10 and 19 years [22], although patients older than 18 were excluded from the study.

The variables included in the statistical analysis comprised background data (the month when infection happened, patient age, gender, place of origin (urban, rural), ethnicity (Romanian, Roma), measles vaccination status (unvaccinated, incomplete vaccination, or complete vaccination when two MMR doses were given), months from last MMR dose, prematurity status (born premature or full-term), nutritional status (poor, good), existence of congenital malformations, and infection source (family, collective, isolated)), complications (conjunctivitis, otitis media, upper respiratory tract infection, pneumonia, acute respiratory failure, thrombocytopenia, sepsis, liver damage, seizure, anemia), signs and symptoms (Koplik’s spots, maculopapular rash, hyperpigmented rash, coryza, fever, cough, diarrhea), antibiotic treatment, symptomatic treatment, length of hospital stay, ICU admission, chest X-ray findings, laboratory parameters (white blood cells, lymphocytes, hemoglobin, platelets, alanine aminotransferase, aspartate aminotransferase, blood urea nitrogen, creatinine, lactate dehydrogenase, procalcitonin, c-reactive protein, fibrinogen), and infection outcome (mortality).

### 2.3. Statistical Analysis

Statistics were performed using MedCalc v.20. The absolute (n) and relative (percent) frequencies of categorical variables were computed, and their proportions were compared using the Chi-square and Fisher’s exact tests. The Mann–Whitney test was used to compare variables that were not Gaussian and were characterized as the median and interquartile range (IQR). The Student’s *t*-test was used to compare the mean and standard deviation of continuous variables with a normal distribution (unpaired, independent samples). A multivariate analysis was computed with the development of pneumonia as dependent variable for both children and adolescent groups. Only the unvaccinated children were included in the regression, and the results were expressed as odds ratio (OR) and confidence interval (CI). The alpha value was set at a significance level of 0.05.

## 3. Results

### 3.1. Background Analysis

During the one-year study period allocated for data collection, a total of 136 measles patients younger than 18 years old were eligible for inclusion in the current analysis. Among the cohort of pediatric patients, there were 104 children younger than ten years old and 32 patients aged between 10 and 18 years old that formed the two comparison groups. The average age in the group of children was 2.4 years, with a statistically significant difference from the group of adolescents with an average of 12.5 years (*p*-value < 0.001). An important characteristic of both study groups was the high prevalence of patients from the Roma ethnicity, which, although representing a minority in Romania, the prevalence was more than 40% in the current study (Table 1).

The infection source was considered as familial, collective, or isolated infection when the epidemiological analysis could not trace the source from a positive contact. A collective source of infection was considered as a community infection with measles that occurred outside the family. A total of 40.4% children had their family as source of infection, while only 21.9% of adolescents got infected from a familial source (*p*-value = 0.047). However, 71.9% of measles infections in adolescents were isolated. The proportion of pediatric patients with a poor nutritional status was significantly higher in the group of children compared with the group of adolescents (30.8% vs. 12.5%, *p*-value = 0.040). Although the majority of patients were unvaccinated against measles (91.9%), there were six patients with an incomplete vaccination status and another four with a complete vaccination profile who still got a symptomatic infection. It was observed that the average duration from the last MMR dose until measles infection was significantly higher in the group of adolescents compared to children (70.2 months vs. 9.6 months, *p*-value < 0.001).

**Figure 1:**
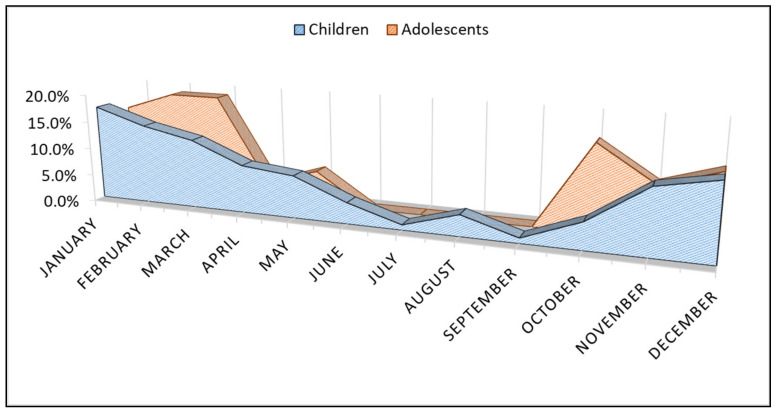
Comparison of measles cases proportions by month of infection with stratification by group of children and adolescents.

The comparison of proportions of measles infections by month between children and adolescents revealed that the highest incidence (>20%) was between October-November, and February-March among adolescents, and November to January among children, as presented in Figure 1.

### 3.2. Clinical Profile and Biological Parameters

**Figure 2:**
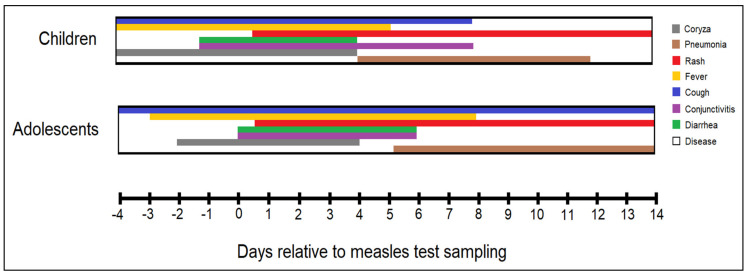
Timeline of measles signs and symptoms occurrence and duration, stratified between children and adolescents relative to measles IgM test sampling. Day 0 is considered the date of IgM antibody testing.

Table 2 describes the clinical characteristics, complications, and outcomes of patients admitted with measles, stratified by patients age as children younger than ten years and adolescents between 10 and 18 years old. Additionally, Figure 2 presents the timeline of measles signs and symptoms between children and adolescents relative to measles IgM test sampling. Koplik’s spots were more commonly observed among adolescents (37.5%) compared to children (20.2%), with a statistically significant difference between groups (*p*-value = 0.045). Besides Koplik’s spots, other hallmarks of measles infection in children, such as conjunctivitis and coryza were observed in approximately 50% and, respectively, 25% of all studied patients, probably due to late presentations when the characteristics of infection are changing. Among complications, children suffered significantly more cases of otitis media (11.5% vs. 0.0%, *p*-value = 0.044), although there were more adolescents with pneumonia and acute respiratory failure (81.3% vs. 60.6%, *p*-value = 0.032), respectively (12.5% vs. 2.9%, *p*-value = 0.031). In consequence, the imaging studies by x-ray or computed tomography revealed more cases with bilateral consolidation appearance among adolescents (*p*-value = 0.045). Furthermore, there were 25.0% adolescents who did not require antibiotic treatment during hospitalization, compared with 10.6% children (*p*-value = 0.039). There were only five patients (3.6%) in the whole cohort of pediatric patients who required ICU admission, and there were no mortality cases.

The biological profile of a complete blood count, liver function tests and inflammatory serum markers of pediatric patients with measles is presented in Table 3. It was observed that the average white blood cell count in children was statistically significantly more elevated than in adolescents (8.8 thousand vs. 5.2 thousand, *p*-value < 0.001). The same observation was made for lymphocytes and platelets. However, the mean values of red blood cells and hemoglobin were significantly decreased in children (3.9 million vs. 4.4 million, *p*-value = 0.040), respectively (10.8 vs. 12.3, *p*-value < 0.001). Although alanine aminotransferase levels were, on average higher than the normal range, the difference among study groups was not statistically significant. Lastly, procalcitonin levels among the inflammatory markers was significantly higher in the group of adolescents (0.9 mg/L vs. 0.5 mg/L, *p*-value < 0.001).

### 3.3. Risk Factor Analysis

**Figure 3:**
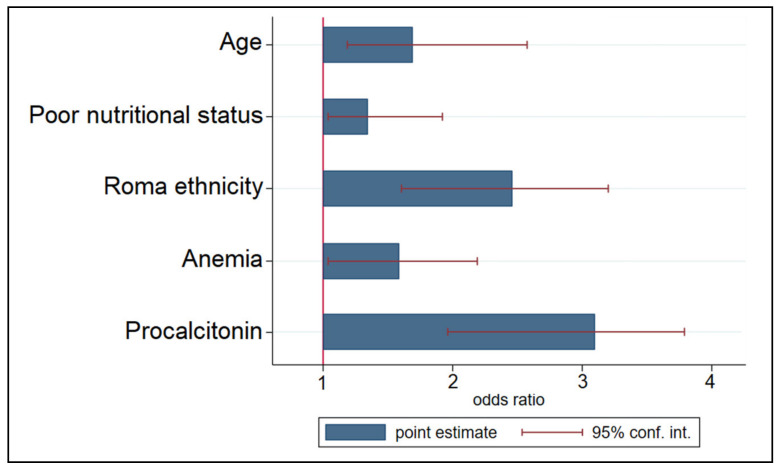
Multivariate risk factor analysis for developing pneumonia in children and adolescent patients with measles.

The multivariate risk factor analysis presented in Figure 3 included a total of 126 unvaccinated children and identified as independent risk factors the age of patients (OR = 1.62), poor nutritional status (OR = 1.25), Roma ethnicity (OR = 2.44), anemia (OR = 1.58), and elevated procalcitonin (OR = 3.09) as statistically significant independent risk factors for the development of pneumonia in pediatric patients with measles.

## 4. Discussion

### 4.1. Current Findings and Existing Evidence

The majority of pediatric patients in this research who had measles presented with a maculopapular rash and a combination of symptoms, including fever and maculopapular rash, and the hyperpigmented rash is a very precise clinical diagnostic tool for measles infection. Therefore, the diagnosis should be made even before confirmation from serology test for anti-measles IgM or measles viral RNA via RT-PCR.

Regarding the measles infection evolution and severity, it was observed that adolescents were more likely to develop pneumonia and secondary bacterial pneumonia, as indicated by significantly higher procalcitonin levels [23,24]. However, the overall number of disease complications, duration of hospital stay and number of patients admitted to the ICU remained insignificant when compared by age groups. It would be expected that the disease evolution to be less aggressive in vaccinated children and adolescents, as found by other studies [25], which is important to consider since the vast majority of our patients were unvaccinated.

It was determined that a poor nutritional status was associated with worse outcomes and a longer duration of hospitalization, similarly to other infections in the pediatric population [26]. Compared with the entire cohort of pediatric patients with measles, those who developed pneumonia were admitted for a significantly longer duration, from a median of 7 days, to a median of 15 days. Generally, it is considered that Romania is among developed countries with a very high Human Development Index (HDI) [27,28], and therefore, malnutrition should not be a concern in the pediatric population, even though we observed a number of 26 children with poor nutritional status, with a high prevalence in the Roma population. Oppositely, in underdeveloped countries such as sub-Saharan countries, it was observed that measles has more severe consequences for children’s health as a result of widespread immunosuppression caused by malnutrition. According to the Demographic and Health Survey conducted in the Democratic Republic of the Congo, 23 percent of children younger than five years old were acutely malnourished, and 43 percent were chronically malnourished [29]. It has been shown that malnutrition is associated with impaired cell-mediated immunity [30] and, in underdeveloped regions, with an increased risk of morbidity and mortality in measles patients, or failure of a proper immune response after vaccination. It was reported in the literature that even though two doses of the MMR vaccine provide a strong long-lasting immunity, still up to 3% of vaccinated individuals get infected with measles, likely due to an improper immune response [31].

Several reports for children mortality caused by measles estimated a measles fatality rate ranging from 5 percent to 25 percent in developing countries [32]; by contrast, a 1 percent mortality rate was seen in the general population of the United States, up to 3% in hospitalized patients during one of the last measles outbreaks before its eradication [33]. In other European countries, measles mortality was reported to be much lower, at around 0.2% of all infections [34]. However, in the current study there were no death reports, likely due to the low number of cases involved.

Apart from that, measles infection, especially in undernourished infants, significantly increases the risk of corneal ulceration from keratitis and consequent blindness. In our children cohort, there were no cases of keratitis, and 29.8% of them developed conjunctivitis. A comprehensive review of complications given by measles and several African studies reported a higher incidence of blindness in malnourished patients with vitamin A deficiency [35,36]. Approximately 15,000 to 60,000 instances of blindness are reported each year among children in low-income countries due to measles blindness, which is the most common cause of blindness among children in this population [37]. It was demonstrated that a strong synergistic relationship exists between measles and vitamin A deficiency, resulting in corneal ulceration, keratomalacia, and eventual corneal scarring or phthisis bulbi. In underdeveloped nations, high-dose oral vitamin A supplementation is advised for all children who have measles and are hospitalized. The most effective measures for preventing measles blindness are increased measles vaccine coverage to halt measles transmission and programs focused on boosting vitamin A in children [38].

As disease complications often require longer hospitalizations, the cost of prolonged treatment should be considered during epidemics. Although we did not estimate the costs of hospitalizations, private healthcare systems are more likely to focus on financials, as a study did on cases of measles that needed inpatient treatment. Based on historical statistics, the Centers for Disease Control and Prevention (CDC) estimates that roughly one in every four cases of measles in the United States results in hospitalization and that one in every 1000 cases leads to death [39]. Hospitalizations for measles dropped sharply after widespread immunization against the disease, and it was recently considered eradicated [40]. In the absence of immunization, there would be around 400,000 hospitalizations costing more than $3 billion USD and more than 1800 fatalities per year [41].

Other treatment options for patients under 18 years old are immunoglobulins. Existing guidelines in the United States recommend measles-specific immunoglobulins (IMIG) administration for children younger than six months and for infants aged six to eleven months who cannot get MMR within 72 h of exposure [42]. A dosage of 0.5 mL/kg to a maximum of 15 mL is advised. Intravenous immune globulins (IVIG) are indicated for immunocompromised contacts unless they are currently getting sufficient IVIG or SCIG as part of their condition’s therapy. IVIG is also indicated for pregnant women who are not immune. The recommended dosage of IVIG is 400 mg/kg, considering that IMIG may be used for additional non-immune contacts within six days of exposure, while MMR is recommended if delivered within 72 h of exposure, and that priority should be given to contacts with close and extended exposure [43].

### 4.2. Limitations and Future Perspectives

The present research integrates critical data on the epidemiology of measles in Romania, its clinical and paraclinical symptoms in the unvaccinated pediatric population, and the factors associated with the development of pneumonia. Thus, several concerns should be addressed. To begin, the sample size was limited, reducing the statistical power of the study. The sample size was also affected by several incomplete patient paper records. Second, the retrospective design of the research is reliant on good patient recordkeeping, as well as the accuracy of data transcribed digitally from paper records. Considering the existence of a significant number of pediatric patients with measles among Roma people, it is of important interest to further study this particular population in comparison with the Romanian population of patients. Lastly, considering the retrospective design of the study, it was not possible for the research team to follow a standard checklist when reviewing the infected patients. Therefore, several patients were excluded from the analysis for incomplete records.

## 5. Conclusions

Measles pneumonia is a life-threatening consequence for measles-infected patients. It was observed that the vast majority of hospitalized pediatric patients with measles were unvaccinated, with an important concern among patients in the Roma community that represented more than 40% of these hospitalized patients. Among these patients, pneumonia was a significant complication, and careful consideration of risk factors such as the age of patients, poor nutritional status, Roma ethnicity, anemia, and procalcitonin levels should be addressed before severe complications occur. Although several treatment options are available for pneumonia, preventive healthcare is imperative. If parents continue to express reluctance to vaccination, physicians should listen to and address any questions or concerns, moreover in the Roma population that has the lowest vaccination rate, this being an excellent chance to educate parents and work toward total eradication via vaccination.

## Figures and Tables

**Table 1:** Comparison of background characteristics of patients admitted with measles by the duration of hospital stay.

Variables	Children (*n* = 104)	Adolescents (*n* = 32)	*p*-Value *
Background			
Age (mean ± SD)	2.4 ± 2.1	12.5 ± 2.2	<0.001
Sex—female	52 (50.0%)	15 (46.9%)	0.757
Born premature—yes	17 (16.3%)	3 (9.4%)	0.330
Place of origin—urban	47 (45.2%)	14 (43.8%)	0.885
Ethnicity—Roma	44 (42.3%)	13 (40.6%)	0.866
Infection source			0.047
Family	42 (40.4%)	7 (21.9%)	
Collective	1 (1.0%)	2 (6.3%)	
Isolated case	61 (58.7%)	23 (71.9%)	
Vaccination status			0.530
Unvaccinated	97 (93.3%)	29 (90.6%)	
Incomplete	3 (2.9%)	3 (9.4%)	
Complete	4 (3.8%)	0 (0.0%)	
Nutritional status			0.040
Poor	32 (30.8%)	4 (12.5%)	
Good	72 (69.2%)	28 (87.5%)	
Months from last MMR dose (mean ± SD)	9.6 ± 6.2	70.2 ± 29.4	<0.001
Congenital immune deficiency	4 (3.8%)	0 (0.0%)	0.363

* Data reported as *n* (%) and analyzed using Chi-square or Fisher’s exact test; Data stratified by patient’s age as children (<10 years old) and adolescents (≥10 years old); SD—Standard Deviation; MMR—Measles Mumps Rubella vaccine.

**Table 2:** Comparison of clinical characteristics, complications, and outcomes of patients admitted with measles, stratified by duration of hospital stay.

Variables	Children (*n* = 104)	Adolescents (*n* = 32)	*p*-Value *
Signs and Symptoms			
Koplik’s spots	21 (20.2%)	12 (37.5%)	0.045
Maculopapular rash	94 (90.4%)	27 (84.4%)	0.342
Hyperpigmented rash	10 (9.6%)	5 (15.6%)	0.342
Fever	101 (97.1%)	30 (93.8%)	0.376
Coryza	50 (48.1%)	16 (50.0%)	0.849
Cough	101 (97.1%)	30 (93.8%)	0.376
Diarrhea	29 (27.9%)	8 (25.0%)	0.748
Complications			
Conjunctivitis	31 (29.8%)	8 (25.0%)	0.598
Otitis media	12 (11.5%)	0 (0.0%)	0.044
Upper respiratory tract infection	27 (26.0%)	11 (34.4%)	0.353
Pneumonia	63 (60.6%)	26 (81.3%)	0.032
Acute respiratory failure	3 (2.9%)	4 (12.5%)	0.031
Thrombocytopenia	4 (3.8%)	1 (3.1%)	0.849
Sepsis	2 (1.9%)	1 (3.1%)	0.685
Liver damage	11 (10.6%)	6 (18.8%)	0.221
Seizure	3 (26.9%)	0 (0.0%)	0.331
Anemia	26 (25.0%)	10 (31.3%)	0.483
Chest X-ray			
Bilateral consolidation	61 (58.7%)	25 (78.1%)	0.045
Interstitial pattern	28 (26.9%)	14 (43.8%)	0.071
Antibiotic treatment			
None	11 (10.6%)	8 (25.0%)	0.039
Cephalosporins	71 (68.3%)	16 (50.0%)	0.059
Clarithromycin	5 (4.8%)	2 (6.3%)	0.746
Penicillins	7 (6.7%)	2 (6.3%)	0.923
Others	10 (9.6%)	4 (12.5%)	0.638
Hospital stay—days (median [IQR])			
All patients	7 (4–9)	7 (4–11)	0.881
Patients with pneumonia			
ICU admission	4 (3.8%)	1 (3.1%)	0.849
Mortality	0 (0.0%)	0 (0.0%)	-

* Data reported as *n* (%) and analyzed using Chi-square or Fisher’s exact test; Data stratified by patient’s age as children (<10 years old) and adolescents (≥10 years old); IQR—Interquartile Range; ICU—Intensive Care Unit.

**Table 3:** Comparison of biological parameters of patients admitted with measles, stratified by duration of hospital stay.

Variables	Normal Range	Children (*n* = 104)	Normal Range	Adolescents (*n* = 32)	*p*-Value *
WBC (thousands/mm^3^)	5.0–15.0	8.8 ± 4.5	4.5–11.0	5.2 ± 2.6	<0.001
Lymphocytes (thousands/mm^3^)	3.0–9.5	5.9 ± 1.7	1.0–4.8	4.7 ± 1.2	0.003
RBC (millions/mm^3^)	4.20–6.10	3.9 ± 1.1	4.35–5.65	4.4 ± 1.2	0.040
Hemoglobin (g/dL)	11.0–13.5	10.8 ± 1.5	13.0–17.0	12.3 ± 1.5	<0.001
Platelets (thousands/mm^3^)	150–350	312 ± 148	150–450	212 ± 122	<0.001
ALT (U/L)	10–40	52.6 ± 33.1	7–35	55.0 ± 40.1	0.731
AST (U/L)	10–35	28.9 ± 19.1	10–40	42.9 ± 16.0	0.076
LDH (U/L)	100–250	251 ± 106	140–280	268 ± 114	0.099
Procalcitonin (ug/L)	0–0.5	0.5 ± 0.3	0–0.5	0.9 ± 0.6	<0.001
CRP (mg/L)	0–14	29.7 ± 14.3	0–10	29.9 ± 15.0	0.989
Fibrinogen (g/L)	2–4	9.1 ± 4.3	2–4	8.9 ± 4.1	0.127

* Data reported as mean ± SD and analyzed using Student’s *t*-test; Data stratified by duration of hospital admission as short stay (less or equal with the median of 6 days), respectively long stay (more than the median of 6 days); WBC—White Blood Cells; RBC—Red Blood Cells; AST—Aspartate Aminotransferase; ALT—Alanine Aminotransferase; LDH—Lactate Dehydrogenase; BUN—Blood Urea Nitrogen; CRP—C-reactive Protein.

## Data Availability

Data available on request.
